# Threat to Professional Autonomy and Physicians' Intention to Use Acupuncture: A Study From Malaysia

**DOI:** 10.3389/fpubh.2022.820786

**Published:** 2022-06-02

**Authors:** Aqil M. Daher, Siew Siew Ong, Devanandhini Krisnan

**Affiliations:** ^1^Department of Community Medicine, School of Medicine, International Medical University, Kuala Lumpur, Malaysia; ^2^Department of Chinese Medicine, Centre for Complementary and Alternative Medicine, International Medical University, Kuala Lumpur, Malaysia

**Keywords:** professional, autonomy, acupuncture, intention, physician

## Abstract

Despite its popularity, registered medical practitioners (RMPs) are reluctant to use acupuncture in their practice. The aim of this study is to determine the impact of Threat to Professional Autonomy (TPA) on RMPs' intention to use acupuncture in Malaysia. A cross sectional study was conducted using an online survey form. The survey was distributed to 250 registered medical practitioners who are affiliated with the Malaysian Medical Association. The questionnaire followed a modified technology acceptance theoretical framework including the three main constructs of ease of use, usefulness and intention to use with addition of TPA as a predictor of physician intention. Structural equation modeling (SEM) was utilized to test the relationship between the 4 constructs. Measurement model, discriminant validity and path analysis statistics were presented. Two hundred and seventeen returned the completed questionnaire yielding a response rate of 86.8%. In the measurement model, all items within each construct were highly correlated. The minimum average variance extracted (AVE) was 0.741. All constructs achieved a minimum of 0.896 reliability estimates. Discriminant validity was ascertained with the findings that the square root of AVE is larger than the correlation between each two constructs. TPA has a significant negative impact on ease of use (*p* < 0.001) and perceived usefulness (*p* = 0.002). There was no significant direct effect of TPA on intention (*p* = 0.0561). Fit indices showed adequate fit. In conclusion, TPA affects the intention to use acupuncture indirectly through its negative effect on perceived ease of use and perceived usefulness of acupuncture.

## Introduction

With increased healthcare cost and focus on patient reported indicators, a new paradigm emerged as an alternative to the traditional pharmacological treatment.

Traditional Chinese Medicine (TCM) takes a holistic approach in treating patients based on individualized treatment utilizing the concept of “Syndrome Differentiation”.

Currently, TCM accounts for 40% of all health services delivered in China (1). Studies from western countries like the USA (2), Japan (3), Denmark (4) and other countries have shown popularity and acceptance of TCM. Among the most popular TCM practices is acupuncture. Systematic review, randomized clinical trials and observational studies have documented the efficacy and utility of acupuncture (5). Reflecting its importance and contribution, the World Health Organization (WHO) recommended the inclusion of acupuncture into healthcare policies (6).

In Malaysia, acupuncture, as part of TCM, was introduced in the fourteenth century. However, with influx of Chinese migrants to Malaysia, the first two hospital providing range of TCM modalities were established in 1894 (7). Following the enactment of Traditional and Complementary Medicine (T&CM) Act 2016, T&CM Council is officially the regulatory authority to govern the practices from self-regulation to statutory regulation regimens (8).

National Health and Morbidity Survey 2015 showed that the prevalence of T&CM use in the preceding 12 months was 29.25% (9). By the year 2017, it was reported that the lifetime prevalence of using T&CM by Malaysians is 69.4% (8).

The importance and contribution of T&CM in Malaysia is reflected by the out-of-pocket expenditure on T&CM treatment. It was estimated that Malaysia spends approximately US$500 million annually on traditional therapies compared to only US$300 million on conventional therapies (10). In tandem with the main T&CM use worldwide for problems related to musculoskeletal system, Malaysian population is also likely to use T&CM for health problems mainly related to myalgia, back pain, join pain and muscle ache (9).

Formal degree in TCM is offered in few Malaysian institutes (11). Moreover, there is a separate governance imposed on the practice of western medicine and the practice of alternative medicine (12). In addition to the mainstream TCM practitioners, some registered medical practitioners (RMPs) like anesthetists and pain management specialists have been performing acupuncture under the credentialing and privileging (C&P) from their hospitals and institutions. However, there are certain quarters of RMPs who have been freely performing this therapy in their day-to-day practice without undergoing due C&P process.

Despite gaining wide acceptance by consumers, there is a reluctance of some medical practitioners to include acupuncture in their practice. This may probably be due to unfamiliarity with techniques; lack of evidence for the effectiveness of this technique; limited time of consultation; access to resources (13) or legal issues (14).

Professional autonomy is defined as professionals' having control over the conditions, processes, procedures, or content of work according to their own collective and, ultimately, individual judgment in the application of their profession's body of knowledge and expertise (15).

Autonomy is a right which is attained by the virtue of membership to a specific profession. Autonomy is conferred based on acquired knowledge, attitude and practice that enables the professional to operate independently and make decisions based on knowledge and expertise (9, 10).

Threat to professional autonomy (TPA) is inherent in medical practice. While clinical practice guidelines (CPG) are aimed to improve patient outcomes, data from South Florida showed that physician resistance to CPG was attributed to concern over loss of autonomy (16).

Similarly, general practitioners from the UK showed resistance to those clinical guidelines when it poses a threat to their autonomy (17). A review of published studies demonstrated that the performance indicators were seen as jeopardy to professional autonomy by practitioners from southern England (18). Around 68% of surveyed physicians from the UK believed that clinical freedom is essential and any constraints to freedom should be fought (19). A Canadian study showed that physicians have continuously resisted the prevailing insurance plan that posed a threat to their autonomy (20).

Studies examining role of TPA among physician are scarce. Findings from 335 Malaysian physician who are working in 12 public and private hospital showed that among other factors, TPA negatively affected the physician's intention to adopt clinical decision support system (CDSS) (8, 21). Data collected from 1,000 randomly selected physicians in the US showed that TPA has a negative direct effect on the physician's intention to use information technology in their clinical decision support systems than for electronic medical records systems (22).

The role of threat to professional autonomy hasn't been examined as an independent factor which may determine the physician's intention to use acupuncture in their practice. Thus, we undertook this study to explore the relationship between threats to professional autonomy, between TPA, ease of use, perceived usefulness, and intention to use acupuncture.

## Methods

### Procedure

A cross sectional study was conducted using an online survey form. The survey was distributed to 250 RMPs who are affiliated with Malaysian Medical Association (MMA). RMPs are medical professionals who treat conditions, symptoms, or diseases using a range of drugs, surgery, or therapies.

The Malaysian Medical Association (MMA) which has a current membership of 10,000 is the main representative body for most of the RMPs in this country. This association's membership is open to all registered medically qualified practitioners possessing valid and recognized basic medical degrees listed in the schedule in the medical act 1971. This includes medical practitioners serving all levels of clinical care i.e. primary, secondary and tertiary. Qualifications range from basic medical degree to that of specialist and subspecialty level. Sampling from this group is ideal as it represents most registered practitioners in Malaysia, irrespective of age, gender, ethnicity, locations (urban/rural) as well as public or private service. All active MMA members with good standing and with email/phone contact were contacted in this study.

### Theoretical Framework and Hypotheses

This study utilized the Technology Acceptance Model (TAM) as basis for investigating the role of threat to professional autonomy in predicting physicians' intention to use acupuncture in their practices. Although TAM is related to application of technology, theoretically it is an extension of the reasoned action theory of planned behavior. TAM has shown its stability in different populations and settings.

The original TAM model stipulates that individual intention (I) to use technology is influenced by the perceived ease of use (PEU) and perceived usefulness (PU) of the said technology. In this study, factors that are assumed to affect physician decision to use acupuncture in practice, beyond the licensing/legal issues, are identical to those of TAM. However, a new dimension, TPA, has been investigated as a possible external predictor.

The following hypotheses were tested.

Hypothesis 1: Perceived usefulness of acupuncture is positively related to physician intention to use acupuncture.

Hypothesis 2: Perceived ease of use of acupuncture is positively related to physician intention to use acupuncture.

Hypothesis 3: Perceived ease of use of acupuncture is positively related to Perceived usefulness of use of acupuncture.

Hypothesis 4: Threat to professional autonomy is negatively related to Perceived ease of use acupuncture.

Hypothesis 5: Threat to professional autonomy is negatively related to Perceived usefulness of acupuncture.

Hypothesis 6: Threat to professional autonomy is negatively related to physician intention to use acupuncture.

### Questionnaire

The questionnaire was developed with reference to the TAM questionnaire. The main constructs which are ease of use, perceived usefulness and intention were adapted from the original TAM (23). TPA questions were adapted from Walter and Lopez (22). Pilot study and exploratory factor analysis showed that each construct is adequately represented by three items which are rated on 5-point Likert scale. In addition, the questionnaire captured basic characteristics like age, gender, qualification, possession of TCM qualification, type of working organization and tier of service.

### Sample Size Determination

Sample size was derived based on the most popular recommendation for studies using SEM. Kline recommended a minimum of 200 cases for incomplex model (21, 24). Using the online calculator which is based on number of indicators in the model, expected effect size and level of significance, sample size was found to be 137 with recommended size of 200 (25). We opted to set the sample size at 250 considering a 25% non-response rate.

### Sampling Method

The list of RMPs registered with MMA with active status was obtained from MMA secretary. Participants were selected with simple random sampling using computer aided program. Selected participants were contacted via email and the online form was sent for completion.

### Statistical Analyses

The data was entered and analyzed using SPSS v.25 software. Categorical variables presented with frequency and percentage. Exploratory data analysis was performed using SPSS v.25 to ensure sample adequacy and sufficient correlations between items. Using the Analysis of Moment Structure (AMOS) software, Structural equation modeling (SEM) was utilized to test the relationship between the 4 constructs. Measurement model was presented with loadings; average variance extracted; Cronbach's alpha and composite reliability. Discriminant validity was ascertained with findings of average variance extracted (AVE) being larger than the between-construct correlations. Standardized regression coefficients were presented along with fit indices.

Missing data was minimal, and no imputation was done.

## Results

Out of the 250 distributed questionnaires, two hundred and seventeen participants returned the completed questionnaire yielding a response rate of 86.8%. More than 42% of respondents were less than 40 years of age. Around 58.5% of them were males. Nearly 60.8% were working in public organizations, and 45.1% in primary level of care. Respondents with specialist qualification constituted 61.8% of the sample. Only 8% of respondents had qualification in TCM training (Table 1).

**Table 1:** Characteristics of the study sample.

**Variable**	***n***	**%**
Age	<40	90	42.1
	40–59	67	31.3
	60+	57	26.6
Gender	Male	126	58.3
	Female	90	41.7
Your work place organization	Public	132	60.8
	Private	85	39.2
You are working in	Primary Center	97	45.1
	Secondary Center	18	8.4
	Tertiary center	100	46.5
Qualifications in TCM/Acupuncture	No	199	92.1
	Yes	17	7.9
Highest medical qualification	Basic medical degree	83	38.2
	Specialist qualification	97	44.7
	Subspecialty qualification	37	17.1

### Measurement Model

Table 2 shows the construct validity and reliability statistics. It is observable that all items within each construct were highly correlated. The loadings which represent the correlation of items to their hypothesized factor ranged between 0.819 and 0.973 for perceived usefulness; between 0.838 and 0.939 for intention; from 0.778 to 0.905 for TPA and between 0.837 and 0.898 for ease of use. The AVE represented the true variance captured by the construct hence reflecting the reliability of the measure. It was highest for perceived usefulness and lowest for ease of use. Nonetheless, the minimum AVE was 0.741. Regarding reliability of the measurement model, composite reliability and Cronbach's alpha statistics revealed that, all constructs achieved a minimum of 0.896 reliability estimates.

**Table 2:** Factor loadings and reliability indices.

**Construct**	**Items**	**Loadings**	**AVE**	**Cronbach's alpha**	**CR**
PU	PU1	0.819	0.854	0.942	0.946
	PU2	0.973			
	PU3	0.972			
I	I1	0.898	0.797	0.920	0.921
	I2	0.939			
	I3	0.838			
TPA	TPA1	0.905	0.744	0.893	0.896
	TPA2	0.898			
	TPA3	0.778			
EU	EU1	0.837	0.741	0.896	0.896
	EU2	0.898			
	EU3	0.847			

*AVE, Average Variance Extracted more than 0.5*.

*CR, Composite reliability more than 0.7*.

Table 3 shows results of discriminant validity in which diagonals represent the square root of AVE or within construct correlation coefficient. It is observable that the square root of AVE for TAP (0.863) was larger than the correlation between TPA and other constructs. Similarly, the square roots of AVE for EU (0.861), PU (0.924) and I (0.893) were larger than the correlation between constructs satisfying the discriminant validity.

**Table 3:** Correlation Matrix and Square Root of the AVE.

	**TPA**	**EU**	**PU**	**I**
TPA	**0.863**			
EU	−0.261	**0.861**		
PU	−0.326	0.503	**0.924**	
I	−0.259	0.653	0.672	**0.893**

*Bold diagonals represent square root of AVE*.

### Path Analysis

Table 4 presents the standardized regression weights. It was found that when TPA increased, the PEU was significantly reduced by 0.25, (*p* < 0.001). Similarly, when TPA increased, the PU was significantly reduced by 0.21, (*p* = 0.002). The PU was found to increase by 0.45 with each unit increase of PEU. Intention to use acupuncture increased by 0.46 with each unit increase of PU (*p* < 0.001). Similarly, the intention to use acupuncture increased by 0.42 with each unit increase of PEU (*p* < 0.001). Nonetheless, there was no significant direct effect of TPA on intention (*p* = 0.0561). Fit indices showed adequate fit with GFI reaching 0.921; NNFI 0.960 and CFI was 0.970. However, ACFI was lower than 0.9 and RESAM 0. 080. The model explained 59% of variation in intention to use acupuncture.

**Table 4:** Structural model.

**Hypothesis**	**Path**	*B* ^*^	**S.E**.	**β** ^†^	***t***	***p*** ^§^	**Label**
H1	PU → I	0.477	0.067	0.431	6.701	<0.001^*^	Supported
H2	EU → I	0.456	0.073	0.408	6.285	<0.001^*^	Supported
H3	EU → PU	0.486	0.077	0.451	6.302	<0.001^*^	Supported
H4	TPA → EU	−0.181	0.055	−0.251	−3.310	<0.001^*^	Supported
H5	TPA → PU	−0.163	0.052	−0.210	−3.152	0.002^*^	Supported
H6	TPA → I	0.086	0.044	−0.107	−1.955	0.051	Not supported

*R^2^ = 0.59*.

* *Unstandardized regression weight*.

*Standardized regression weight*.

† *t-test value*.

§ *p-value is significant at 0.05*.

## Discussion

Factors affecting physician willingness to apply acupuncture in their daily practice are recognized in the literature. To the best of our knowledge, we are reporting the results of the first study addressing the role of TPA as a predictor of acupuncture use.

The results of this study showed that TPA negatively affects the EU and PU among a sample of RMPs in Malaysia. Thus, it provides support to our hypothesis and offers new evidence to understand physician's intention to apply acupuncture.

The results of this study highlight the application of the TAM theoretical model among RMPs. The model showed adequate fitness achieving a good measurement model with discriminant validity (26). The model has shown a good reliability with internal consistency measure and composite reliability higher than the stipulated threshold (27). Moreover, the findings that square root of AVE is higher than the between construct correlation is a strong indication of discriminant validity (28).

Published reports have supported the ease of use of acupuncture as an important predictor of its applicability in medical practice (29). Being a minimally invasive technique associated with patients' aversion to needles, doctors find it easy to use (13).

The usefulness of acupuncture in different medical fields has been documented in a plethora of literature. Beside its main application in treating pain (30); musculoskeletal conditions (31); it also has been applied in the treatment of menstrual pain (32); cancer patient (33) pregnancy (34); and pediatric field as well (35).

Since this study is the first of its kind, comparison of results to other reports is rather impossible. Nonetheless, TPA was found to have a negative effect on PU of IT among physicians (22) which goes, in some way, along with our results.

Medical practitioners are trained to manage a variety of conditions efficiently within the realm of modern medicine. Their professional autonomy is satisfied by the ability to perform and evaluate those complex tasks (36). Clinical freedom is the most obvious type of professional autonomy; it refers to the ability of doctors to provide care to a patient without being limited by organizational procedures, financial concerns, performance measurement systems, or managerial control (37).

Professional autonomy of physicians is integral to the provision of patient care and to a well-functioning health care system. It allows physicians to make decisions that best meet the needs of patients. Additionally, it allows physicians to advocate for the well-being of patients and society, and to hold each other accountable to ensure the provision of the highest standard of health care (38).

Acknowledging patient autonomy is the paramount principle of modern medicine; the power of making medical decisions granted by professional autonomy is translated as beneficence. No conflict could be viewed between professional autonomy and patients' autonomy unless the physician believes that there is a necessary intervention to be offered utilizing principles of paternalism and in paediatric. Central to this conflict is a moral concern of identifying which disease entities warrant paternalism over patient autonomy.

Nonetheless, health services that are highly appreciated by society are perceived safe when referring to safety studies documented in published reports. However, this may not apply in the Malaysian context due to lack of such information. As such the professional autonomy of RMPs might be threatened when recommending or using a technique that is not fully licensed or approved for their practice (38).

Indisputably, the practice of acupuncture cannot be fully explained by a biomedical model, especially when it comes to severities and priorities. This does pose a dilemma in professional autonomy in ensuring the highest standards of health care. In many ways this might be due to inadequate regulations, guidelines, and licensing procedures.

Prior to the implementation of the new T&CM legislation, practitioners could chose to register with the Health Ministry on a voluntary basis with some 13,000 professional and non-professional practitioners having registered so far (9). Official figures on the exact number of practitioners in Malaysia are not available but it is believed to be “substantial”, with many of them not possessing the required qualifications and credentials.

Consequently, the move from Ministry of Health, Malaysia to establish T&CM council is to ensure professional governance. The main objective is to ensure that T&CM practices in Malaysia are safe and carried out ethically. To obtain T&CM registration, those practitioners have to complete minimum modules among others, the importance of patient confidentiality, medical ethics and hygiene, as well as exams to attest to their competency (39).

In this regard, the outcry of RMPs to lawfully practice acupuncture in Malaysia became more intense in the wake of clinical freedom to exercise professional judgment in the care and treatment of their patients grounded on the principle that care decisions are aimed at promoting the patient's well-being. There are RMPs currently practicing acupuncture under the credentialling and privileging in secondary healthcare with less or no formal education trainings. A quarter of RMPs as general practitioners are performing the acupuncture treatments in their private practice.

The interplay of professional autonomy and RMPs eligibility to legally practice acupuncture, with the required standard of competency remains debatable, let alone the western medicine professional governance body to allow dual registration of medical practitioners who opt to use acupuncture in their practice. Under the current Medical Act 1971, RMPs are allowed to perform evidence based-medicine (40).

As such, our sample might have felt threatened by adopting acupuncture as non-standard practice; acupuncture is part of the ancient practice of Traditional Chinese Medicine (TCM) that requires special training underpinned by TCM philosophy. The new T&CM council stipulates that all practitioners are required to register under the new legislation effective 1st March 2021. Currently, RMPs with TCM undergraduate degree are eligible to obtain practitioners' registration with the council. Such legislation may add pressure to the RMPs autonomy.

Moreover, the possession of autonomy necessitates a conducive environment that facilitates application of skills. Our sample may lack the proper knowledge or attitude toward integration of acupuncture to their practice thus the perceived threats.

Other factors affecting professional autonomy cited in the literature include ideology. The ideology as explained by Plamentaz 1971, refers to a set of beliefs, values and attitudes shared by a group (7). Those ideologies are not independent of judgment. As such, social norms and political environment are perceived to influence ideologies. It is apparent that addressing TPA is challenging and requires in depth investigation.

Despite efforts to report bias-free results, some limitations were inevitable. Firstly, using online survey might be associated with loss of follow up as email identifier wasn't accepted. This affects the possibility of reaching out to the participant in case of missing information. Internet savvy practitioner would respond favorably to the online survey. Nonetheless, it wasn't avertable due to restrictions imposed by Covid-19 pandemic. Secondly, data from non-respondents were unavailable. We couldn't identify those who received the email and opted not to respond. Thirdly, factors affecting TPA were not investigated in this study. It is plausible that participants have different perception of why the professional autonomy was threatened with use of acupuncture. Among other pitfalls of this research is the missing information about ethnicity of the participants. The multi-ethnic and multicultural character of the TCM practitioners in Malaysia would affect the choice of specific RMP based on their ethnic identity. Other identifiable limitation of this study was lack of information on the indication for use of acupuncture. Similarly, lack of information on types of speciality hinders describing use of acupuncture by speciality; it was reported that anesthetist and pain management specialist would be the most frequent users of acupuncture. Finally, despite the significant findings of this study, the generalization is limited to RMPs who are affiliated with MMA only.

In conclusion, TPA is a significant predictor of RMPs' intention to use acupuncture through its indirect negative effect on the ease of use and perceived usefulness of acupuncture. Further studies are required to understand the causes of perceived threat to professional autonomy.

## Data Availability Statement

The raw data supporting the conclusions of this article will be made available by the authors, without undue reservation.

## Ethics Statement

The study was approved by the International Medical University Ethics Committee with Reference Number 4.13/JCM-209/2020. The patients/participants provided their written informed consent to participate in this study.

## Author Contributions

AD designed the study, analyzed data, and wrote the manuscript. SO designed the study, supervised data collection, and wrote the manuscript. DK designed the study, collected the data, and wrote the manuscript. All authors contributed to the article and approved the submitted version.

## Funding

This work was supported by a grant from International medical university with grant number of MSAC I/2020(03).

## Conflict of Interest

The authors declare that the research was conducted in the absence of any commercial or financial relationships that could be construed as a potential conflict of interest.

## Publisher's Note

All claims expressed in this article are solely those of the authors and do not necessarily represent those of their affiliated organizations, or those of the publisher, the editors and the reviewers. Any product that may be evaluated in this article, or claim that may be made by its manufacturer, is not guaranteed or endorsed by the publisher.
